# Research on the Influence Mechanism of Thermal Load on the Au-Sn Sealing Weld State on Three-Dimensional DPC Substrates

**DOI:** 10.3390/ma18153678

**Published:** 2025-08-05

**Authors:** Heran Zhao, Lihua Cao, ShiZhao Wang, He Zhang, Mingxiang Chen

**Affiliations:** 1 School of Mechanical Science and Engineering, Huazhong University of Science and Technology, Wuhan 430074, China; 13998898346@163.com; 2 The 47th Institute of China Electronics Technology Group Corporation, Shenyang 110135, China; 3 Liaoning Key Laboratory of Flexible Electronic Micro-Nano Integrated System, Shenyang 110135, China; 4 Institute of Metal Research, Chinese Academy of Sciences, Shenyang 110016, China; 5 School of Electronic Science and Technology, Hainan University, Haikou 570228, China; sz_wang126@126.com; 6 The 13th Institute of China Electronics Technology Group Corporation, Shijiazhuang 050051, China; 15633085087@163.com

**Keywords:** DPC substrate, gold–tin eutectic, gold–tin–nickel ternary compound, interfacial compound, aging, reliability

## Abstract

Direct copper-plated ceramic (DPC) substrates have emerged as a favored solution for power device packaging due to their unique technical advantages. AuSn, characterized by its high hermeticity and environmental adaptability, represents the optimal sealing technology for DPC substrates. Through the application of vacuum sintering techniques and adjustment of peak temperatures (325 °C, 340 °C, and 355 °C), the morphology and composition of interfacial compounds were systematically investigated, along with an analysis of their formation mechanisms. A gradient aging experiment was designed (125 °C/150 °C/175 °C × oxygen/argon dual atmosphere × 600 h) to elucidate the synergistic effects of environmental temperature and atmosphere on the growth of intermetallic compounds (IMCs). The results indicate that the primary reaction in the sealing weld seam involves Ni interacting with Au-Sn to form (Ni, Au)_3_Sn_2_ and Au_5_Sn. However, upon completion of the sealing process, this reaction remains incomplete, leading to a coexistence state of (Ni, Au)_3_Sn_2_, Au_5_Sn, and AuSn. Additionally, Ni diffuses into the weld seam center via dendritic fracture and locally forms secondary phases such as δ(Ni) and ζ’(Ni). These findings suggest that the weld seam interface exhibits a complex, irregular, and asymmetric microstructure comprising multiple coexisting compounds. It was determined that Tpeak = 325 °C to 340 °C represents the ideal welding temperature range, where the weld seam morphology, width, and Ni diffusion degree achieve optimal states, ensuring excellent device hermeticity. Aging studies further demonstrate that IMC growth remains within controllable limits. These findings address critical gaps in the understanding of the microstructural evolution and interface characteristics of asymmetric welded joints formed by multi-material systems.

## 1. Introduction

Moore’s Law is driving the miniaturization of chips, which is currently steering the innovation of semiconductor technology. It is also deeply integrated with advanced technologies such as three-dimensional integration (3D-IC) and system-in-package (SiP), propelling devices to continuously evolve toward integration, miniaturization, and high power density. As the performance of electronic systems is enhanced, the thermal dissipation power per unit volume increases exponentially. This has led to concentrated thermal stress and interface delamination failures, posing core challenges to achieving high-reliability packaging. Especially in fields such as radar components, deep-sea exploration, aerospace, new energy vehicles, and 6 G millimeter waves, where devices are subjected to prolonged exposure to high-temperature environments (>150 °C), higher demands are placed on the mechanical integrity and thermal stability of the packaging structures.

Direct copper-plated ceramic (DPC) substrates have emerged as a prominent solution for power device packaging, leveraging their unique micro-nano manufacturing process and the synergistic benefits of their material system. In contrast with traditional ceramic substrates, DPC substrates utilize photolithography and electroplating processes to achieve sub-micron precision patterning on the surface of ceramic matrices (such as Al_2_O_3_ and AlN). They boast high thermal conductivity (>170 W/m·K), a low coefficient of thermal expansion (CTE) with excellent matching properties, and substantial current-carrying capacity [1,2].

It is worth noting that one of the key features of DPC substrates is their ability to construct dam structures [3]. This allows them to be integrated with high-reliability packaging technologies, effectively isolating environmental factors such as moisture and oxygen from the chip and providing a long-term, stable, airtight environment. Traditional ceramic substrates predominantly utilize parallel seam welding due to its low cost and high efficiency. However, this process is limited by the shape and size of the substrate and inherently has drawbacks such as poor resistance to salt mist and corrosion, restricting its application in high-humidity and high-corrosion environments. More importantly, parallel seam welding forms spot welds by concentrating instantaneous high power, which is incompatible with the high thermal conductivity copper dam structure of DPC substrates. Driven by advancements in DPC (Direct Plated Copper) technologies, including irregular-shaped substrates, ultra-low dam barrier substrates, and DPC-DPC stacked substrates, Au-Sn eutectic soldering represents the optimal sealing solution for DPC substrates due to its superior hermeticity and adaptability. The morphology, composition, distribution, and evolution behavior of the AuSn welding interface have a decisive impact on the interface’s mechanical strength and thermal fatigue life. Research indicates that under environmental thermal stress loads, the coarsening of intermetallic compounds (IMCs), element segregation, and the initiation of microcracks significantly weaken the interface bonding force, leading to sealing failure. It is important to highlight that the copper dam structure and high-density copper coating features of the DPC substrate have significantly altered the metal-ceramic volume fraction within the package. Traditional ceramic substrates typically exhibit a metal volume fraction of less than 10%, whereas three-dimensional DPC substrates demonstrate a metal volume fraction exceeding 50%. This substantial modification in material distribution necessitates a thorough reassessment of thermomechanical reliability during the product design phase, with specific attention to the critical Au-Sn hermetic sealing interface in three-dimensional DPC substrates. Currently, there is a lack of systematic research on the multiscale characterization and dynamic evolution mechanisms of IMCs at the DPC/Au-Sn interface. Therefore, elucidating the formation and evolution mechanisms of interfacial compounds under thermal stress is of critical engineering significance for improving the service life of power devices in complex operating conditions.

The eutectic transformation temperature of AuSn solder is 280 °C, and its microstructure is predominantly composed of the hexagonal close-packed ζ’ phase (Au_5_Sn) and δ phase (AuSn), with δ-AuSn [4] being the first phase to crystallize during solidification. The presence of Ni plating introduces additional complexity to the reaction process. In the Au–Ni–Sn system, Ni_3_Sn_2_ is the stable intermetallic phase formed during solidification [5]. Certain binary phases in this system exhibit high solubility for the third element. Due to the similar chemical and physical properties of Au and Ni, Ni atoms can substitute Au atoms in the δ-(AuSn) and ζ’-(Au_5_Sn) lattices, forming δ(Ni) and ζ’(Ni) phases. Conversely, Au atoms tend to occupy Ni sublattice positions in Ni_3_Sn_2_ [6], resulting in the formation of a (Ni, Au)_3_Sn_2_ intermetallic compound (IMC) layer at the interface with the same crystal structure. M.O. Alam et al. [7] suggested that the difference in atomic size between Au and Ni induces inherent lattice strain. Yoon et al. [8] investigated the interfacial reaction between AuSn solder and Ni under bump metallization (UBM) and found that only the (Ni, Au)_3_Sn_2_ IMC layer was formed at the interface after reflow at 300 °C. In the Au-Sn/Ni joint aged at 250 °C for 500 h, the growth of a (Au, Ni)Sn IMC layer [6] was observed at the interface, which was found to consist of two distinct sublayers: an Au-rich upper layer and a Ni-rich lower layer. Wang Meng et al. [9] reported that when the brazing temperature was set at 300 °C and held for 90 s, a fine layered eutectic microstructure composed of Au_5_Sn and AuSn was formed within the Au-Sn/Ni solder joint, accompanied by the precipitation of elongated needle-like (Ni, Au)_3_Sn_2_ intermetallic compounds. During subsequent solid-state aging at 200 °C, composite δ(Ni) and ζ’(Ni) intermetallic layers gradually formed at the solder/substrate interface [10]. Xiao-feng Wei et al. [5] studied the diffusion behavior under annealing conditions and confirmed that the (Ni, Au)_3_Sn_2_ compound is structurally based on the Ni_3_Sn_2_ phase [11], albeit with significant Au dissolution. The thickness of the (Ni, Au)_3_Sn_2_ IMC layer increases progressively with aging time [12]. Z.X. Zhu et al. [13] investigated the microstructural evolution of AuSn/Ni welds and found that with prolonged aging, AuSn gradually disappears, and the dominant reaction involves the interaction between Ni and AuSn, leading to the formation of (Ni, Au)_3_Sn_2_ and Au_5_Sn intermetallic compounds.

The three-dimensional DPC substrate achieves hermetic packaging by using Au-Sn solder. To date, systematic investigations into the microstructural evolution and interfacial characteristics of asymmetric welding joints formed through multi-material system coupling remain scarce. This technological gap presents significant challenges for the reliability assessment and optimization of this packaging process. In this study, a sealed cavity is constructed using a three-dimensional DPC substrate and a prefabricated Au-Sn solder cover plate. The formation mechanisms of multiple ternary compounds at the welding interface are explored, along with the phase transformation reaction mechanism during welding. The influence of sealing peak temperature on weld seam state is analyzed in terms of weld morphology, thickness, interface compound formation, and Ni diffusion behavior. Additionally, the correlation between aging conditions and IMC thickness is examined, and the hermeticity of the three-dimensional DPC substrate is comprehensively evaluated.

## 2. Materials and Methods

### 2.1. The Structure of the Welding System

**Figure 1:**
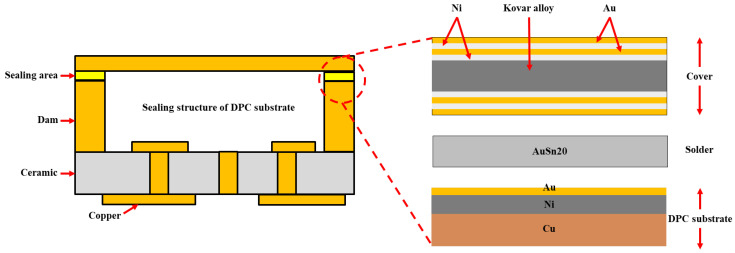
Schematic diagram of the welding system.

Figure 1 illustrates the DPC substrate, solder, and cover welding system, which is composed of three key components: the cover, solder, and substrate. The cover features the most intricate structure, with four sequentially deposited layers—Ni/Au/Ni/Au—having thicknesses of 1.3–8.9 μm, 1 μm, 5 μm, and 0.6 μm, respectively. The outermost Au layer rapidly dissolves into the molten solder, reducing the surface tension of the liquid Au-Sn alloy and promoting its spreading across the cover, thereby ensuring excellent wetting conditions. The Ni layers function as both an effective diffusion barrier to prevent element migration and active participants in interfacial reactions that form high-strength intermetallic compounds. The copper dam of the DPC substrate typically features a Ni/Au double-layer plating structure, where the Au layer improves wettability and the Ni layer serves as an effective barrier to prevent Cu diffusion into the weld seam.

### 2.2. Process Methodology

**Figure 2:**
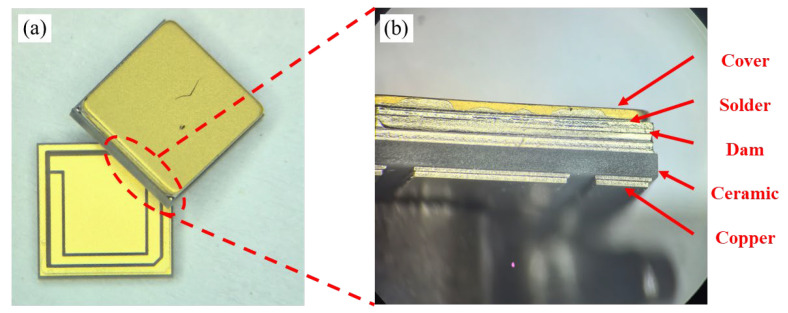
Three-dimensional DPC substrate featuring a gold-tin soldering joint: (**a**) weld seam measurement line and (**b**) element line distribution and side view of the weld seam.

A vacuum sintering furnace was employed as the experimental apparatus. By maintaining a welding pressure of 3.5 N, multiple sets of samples were fabricated by adjusting the sintering peak temperatures to 325 °C, 340 °C, and 355 °C, respectively. Each peak temperature was sustained for 3.5 min. During the preheating phase of the sintering process, the vacuum pump was activated to execute three vacuum cycles in order to eliminate residual air from the chamber. Subsequently, the furnace was purged with high-purity nitrogen (purity exceeding 99.99%) to establish an inert welding atmosphere. This procedure resulted in the formation of Au-Sn sealed DPC dam plate samples, as depicted in Figure 2. Following the completion of sample processing, the microstructure of the welding interface was examined using ZEISS Gemini SEM 460 (ZEISS, Oberkochen, Germany) and subsequently analyzed.

To achieve a comprehensive understanding of the microstructure and phase composition of the bonding interface, the samples were embedded in resin following preparation and subsequently ground and polished until the AuSn weld cross-section (as illustrated in Figure 1) was fully exposed. The interfacial microstructure between the Ni/Au/Ni/Au metallization layers and the AuSn solder was then characterized using SEM. The non-equilibrium solidification morphology of the weld, the evolution of ternary intermetallic compound compositions under different peak temperature conditions, and the thermodynamic driving mechanisms and elemental concentration gradients were systematically analyzed and interpreted. Furthermore, to investigate the correlation between weld evolution, IMC growth, and aging conditions, comparative aging experiments were conducted under both air and argon atmospheres at temperatures of 125 °C, 150 °C, and 175 °C.

## 3. Results

### 3.1. Morphological Characteristics of Non-Equilibrium Solidification in the Weld Seam

**Figure 3:**
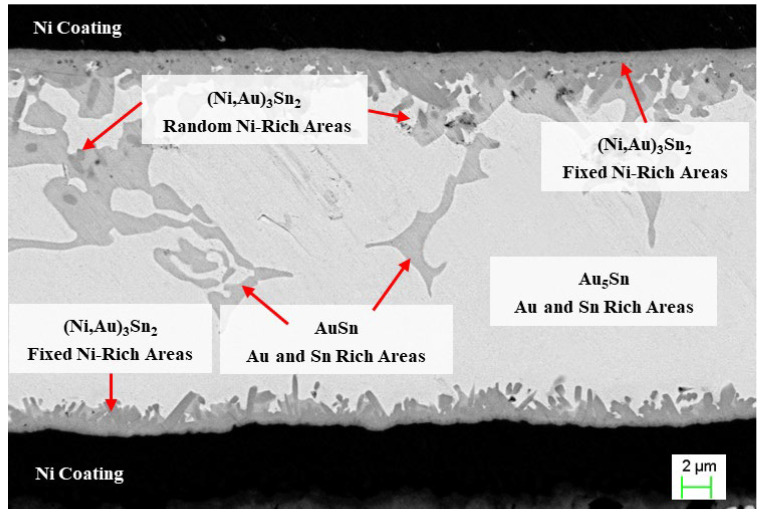
Schematic diagram of the compound and element enrichment zones.

As shown in Figure 3, the vertical cross-section of the cover plate-solder-DPC substrate welding interface obtained through longitudinal grinding reveals the spatial heterogeneity evolution characteristics of the coating structure and intermetallic compounds (IMCs). At the upper end of the weld seam, the original Ni/Au/Ni/Au multilayer coating system is reconfigured into a three-layer structure of Ni/Au/Ni during the sealing process, with the surface Au layer completely dissolving into the molten solder. This confirms that Au acts as an active wetting agent in the interface formation reaction. In contrast, at the lower end of the weld seam, only the Ni layer remains in the Ni/Au coating system, suggesting that this region has experienced a more pronounced Au diffusion-dissolution process. The asymmetric morphology of the weld seam is primarily attributed to the three-dimensional asymmetric packaging structure.

Unlike the “dual-phase continuous interpenetration” morphology observed in typical Au-Sn eutectic solder, the central region of the weld seam exhibits a phase distribution that significantly deviates from the equilibrium eutectic structure. Specifically, the bright white Au-rich phase dominates and forms a continuous matrix, while the dark gray Sn-rich phase is dispersed irregularly within the Au-rich phase as granular particles, discrete isolated islands, or locally connected networks. The interfaces between these two phases are sharply defined, with steep composition gradients and distinct differences in morphology. This non-equilibrium solidification structure demonstrates mutual dispersion of the two phases, with their positions being unpredictable. The formation of this structure can be attributed to three primary factors: (1) the non-uniform spreading of molten solder induced by the three-dimensional geometric constraints imposed by the DPC substrate and cover; (2) asymmetric diffusion of elements between the Ni/Au coating and the solder; and (3) random redistribution of solutes driven by intense thermal motion during the molten state.

Notably, at the interface between the Ni coating and the solder at both the upper and lower ends of the weld seam, a layer of IMCs with uniform thickness is formed. This layer exhibits a continuous and uniform layered structure, accompanied by dendritic branches extending into the weld seam. This phenomenon suggests that during the solid–liquid interface reaction process, the Ni coating acts effectively as a diffusion barrier. Furthermore, the rapid consumption of Au at the interface drives the formation of a multi-level microstructure through dendritic bifurcation, thereby achieving compositional compensation.

### 3.2. Evolution of Ternary Compound Composition and Thermodynamic Driving Mechanism

Based on EDS analysis, it is evident that a deep gray compound forms in the solder region, identified as Au_5_Sn. This phase dominates the bright white main area of the weld seam. Additionally, a small amount of light gray material, identified as AuSn, is observed within the weld seam. The two compounds formed in the weld seam area represent typical AuSn eutectic products (AuSn and Au_5_Sn).

Quantitative EDS analysis reveals that the reaction products at the welding interface exhibit significant ternary alloying characteristics. The main area of the weld seam is predominantly composed of the bright white Au5Sn phase, with localized regions of the light gray AuSn phase, which are characteristic components of the AuSn eutectic phase. The intermetallic compound (IMC) layer at the interface is primarily composed of the deep gray (Ni, Au)_3_Sn_2_ phase, whose crystal structure is inherited from the Ni_3_Sn_2_ parent phase. In this phase, Au atoms substitute into the Ni sublattice via solid solution [12], forming the (Ni, Au)_3_Sn_2_ solid solution. The dendrites that detach into the weld seam retain the composition of (Ni, Au)_3_Sn_2_. Table 1 lists the detection values at several points used to confirm the molar ratios of the compound elements.

In the (Au, Ni)-Sn system, the Ni_3_Sn_2_ phase exhibits Au solubility, while the AuSn phase demonstrates Ni solubility [14]. Although Ni and Au have similar lattice structures, Ni preferentially reacts with Sn at the interface due to the significantly lower formation free energy (∆G) of Ni-Sn compounds compared to Au-Sn compounds [∆G (Ni, Au)_3_Sn_2_ = −93 kJ/mol, ∆G (Au_5_Sn) = −28 kJ/mol]. Generally, it is believed that the possible product of the ternary compound of Ni, Au, and Sn might be (Ni, Au)_3_Sn_4_, as (Ni, Au)_3_Sn_4_ has a lower formation free energy [∆G (Ni_3_Sn_4_) = −135 kJ/mol] than (Ni, Au)_3_Sn_2_. However, in the Au-Sn/Ni system formed by the DPC frame substrate and Au-Sn, the high Ni concentration near the interface limits (Ni, Au)_3_Sn_4_ from becoming the primary reaction product. According to the Au-Sn-Ni ternary phase diagram [5,14], when the Ni content is 43%, the reaction product is Ni_3_Sn_4_, and when the Ni content reaches 60%, the precipitate is Ni_3_Sn_2_. During the short melting and welding process, the limited diffusion distance of Ni restricts the formation of (Ni, Au)_3_Sn_2_ to areas close to the interface.

Notably, the Ni layer exhibits dual functional characteristics in the interface reaction. It acts as a barrier layer to significantly inhibit the cross-interface diffusion of Cu, Fe, Au, and Sn, while also serving as a reactant to selectively combine with Sn. This selective reaction leads to an increase in Au concentration in the interface region, driving the thermodynamically more stable Au_5_Sn phase to grow preferentially. Ultimately, this results in the formation of a composite structure consisting of Au5Sn and (Ni, Au)_3_Sn_2_.

### 3.3. Analysis of the Gradual Change Trend in Element Concentration

**Figure 4:**
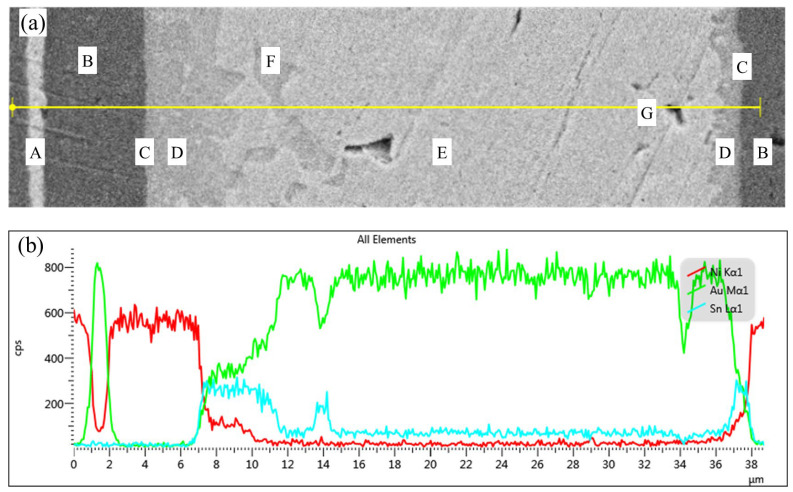
Line scan results of weld seam element composition: (**a**) Weld seam measurement line and (**b**) element line distribution. Red represents Ni, green represents Au, and blue represents Sn.

As shown in Figure 4, the elemental distribution of the Au-Sn-Ni ternary system, analyzed using combined SED and EDS line scanning, exhibits a pronounced spatial gradient. At the interface between zone A (Au coating) and zone B (Ni coating), the Au/Ni concentration gradient demonstrates a stepwise attenuation, confirming that the Ni layer serves as an effective diffusion barrier to inhibit Au diffusion. Near the interface in zone B, the Ni content reaches up to 98.01%, indicating its strong blocking effect on Sn diffusion.

In zone C (interface reaction zone), there is a dramatic change in elemental distribution: the Ni concentration sharply decreases while Au gradually enriches, and the Sn concentration instantaneously peaks. This behavior can be attributed to the dissolution of surface Au by molten solder and the limited diffusion of Ni into the solder, forming a localized Au-Sn/Ni ternary reaction system.

Within zone D (IMCs layer), the Ni concentration decays exponentially, while Sn continuously enriches, confirming that the Ni-Sn alloying reaction dominates the phase selection at the interface. The increasing distribution of Au in this region indicates its partial involvement in the formation of the (Ni, Au)_3_Sn_2_ solid solution; however, the substitution degree of Au is constrained by Ni’s high affinity for Sn.

In zone E (weld center), the Ni concentration approaches zero, the Sn content significantly decreases, and Au becomes dominant. This confirms that the diffusion limitation of Ni leads to the formation of a single-phase Au_5_Sn structure in the weld center, with no δ (Ni) or ζ’ (Ni) substitution phases detected. This phenomenon arises from the selective consumption of Sn during the interface reaction, causing the remaining Sn/Au molar ratio to deviate from the eutectic composition and inhibiting the formation of AuSn phases. F represents the minor presence of the AuSn phase in the weld center region. The concentration variation in zone G (sealing void) is attributed to unstable detection caused by the interruption of the interface.

Notably, the Sn concentration exhibits a three-peak distribution, with the primary peak located at the interface and secondary peaks observed toward the center. This reflects the exponential decay of Ni’s competitive capture effect on Sn with increasing diffusion distance. The Ni concentration shows a double-peak distribution near the interface. This concentration gradient evolution reveals that the limited diffusion depth of Ni and the competitive distribution of Sn jointly regulate the composition of the interface IMCs phase.

## 4. Discussion

### 4.1. Dynamic Phase Transformation Reaction Mechanism During the Sealing Process

During the sealing process, the AuSn solder system experiences an unbalanced thermal cycle (heating to 330 °C followed by cooling to room temperature), and its phase transformation exhibits multi-stage dynamic characteristics. In the heating stage, eutectic melting at 280 °C induces rapid dissolution of the Au coating, forming an Au-enriched zone at the interface and diffusing toward the center of the molten pool. Subsequently, the Ni layer is exposed to the molten solder. Its low dissolution rate effectively suppresses corrosion of the substrate and cover base materials while enabling Ni to diffuse toward the interface and accumulate there.

In the cooling stage, the interface and weld seam center demonstrate distinct phase selection behaviors. At the interface, the Ni-Sn reaction dominates, generating dendritic (Ni, Au)_3_Sn_2_ whose growth is co-regulated by the thermal gradient and solute redistribution. In the weld seam center, the Au-Sn eutectic path is followed: the metastable ζ phase forms first and then transforms into the stable ζ’ phase (Au_5_Sn) and δ phase (AuSn) via a peritectic reaction (L + ζ → ζ’ + δ) below 190 °C. The competitive Sn capture by Ni causes an imbalance in the Sn/Au molar ratio of the solder, driving the system to compensate for compositional deviation through Au_5_Sn precipitation. This ultimately results in a microstructure predominantly composed of bright white Au_5_Sn with minor contributions from light gray AuSn. It is noteworthy that trace amounts of Ni, accompanying the dendrites entering the weld center region, contribute to the formation of secondary phases, specifically (Ni, Au)_5_Sn and (Ni, Au)Sn. The volume fractions of these phases are regulated by the peak temperature and holding time.

### 4.2. Regulation Mechanism of Peak Temperature on IMC Morphology

The interfacial intermetallic compounds exhibit a uniform and continuous layer-like morphology. As the peak temperature increases, undulating and dendritic intermetallic compounds gradually grow on the layer-like compounds, demonstrating a significantly enhanced tendency to expand into the weld seam. The diffusion range into the weld seam increases markedly with rising temperature. With increasing peak temperature, the interfacial intermetallic compounds formed between the Ni coating at the lower end of the weld seam and the AuSn solder also show an increased tendency to expand into the weld seam; however, the diffusion range remains relatively low.

**Figure 5:**
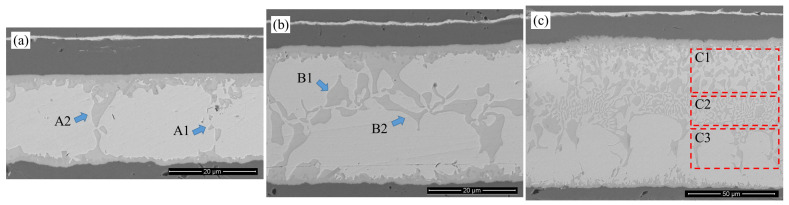
Morphology of AuSn hermetic welds at different peak temperatures: (**a**) 325 °C; (**b**) 340 °C; (**c**) 355 °C.

Based on the SEM morphology analysis presented in Figure 5, the AuSn eutectic weld seams form dense metallurgical bonds within the peak temperature range of 325–355 °C, but the microstructure exhibits significant temperature dependence.

Specifically, the morphology evolution of the interfacial IMCs shows a pronounced temperature gradient dependence. At Tpeak = 325 °C, the light gray phase in the center of the weld seam presents a dual-scale structure. In the A1 region, nanoscale discrete phase blocks with dimensions of 1.26 μm × 1.26 μm are observed; in the A2 region, these coarsen into large-sized phase islands measuring 5.49 μm × 9.60 μm. When the peak temperature is raised to 340 °C, the volume fraction of the light gray phase in the weld seam increases, and its morphology transitions into a competitive growth mode characterized by blocky and strip-like structures. In the B1 region, the size is 6.10 μm × 7.47 μm, similar to the A2 region; in the B2 region, it grows in a strip-like manner with a minimum width of 1.14 μm and an aspect ratio of 11.2:1. Further, at Tpeak = 355 °C, the proportion of the light gray phase in the weld seam increases substantially, dispersing continuously from the upper end to the lower end of the weld seam with significant directionality. This results in a three-level stratified structure: in the interface C1 region, the typical size of the blocky compound is 4.10 μm × 5.24 μm. In the center C2 region of the weld seam, the minimum width of the strip-like compound is 0.95 μm, with a typical aspect ratio of 17.8:1, which is more dendritic compared to the B2 region. In the C3 region beyond the center, the compound islands exhibit a transitional morphology between blocky and strip-like, with a lateral span of 9.14 μm and a longitudinal span of 21.14 μm.

**Figure 6:**
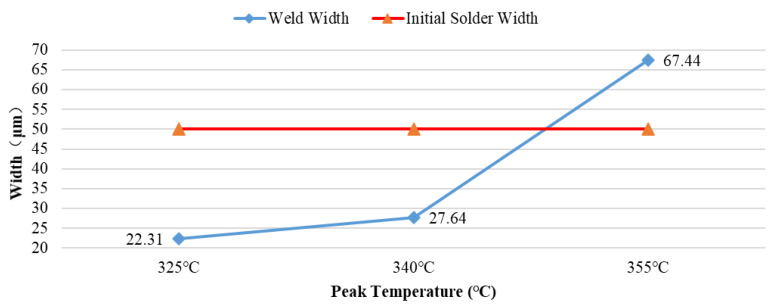
Relationship between weld thickness and peak welding temperature.

The results further indicate that the thickness of the Au-Sn eutectic weld seam exhibits a significant nonlinear evolution characteristic with respect to the sealing peak temperature (Tpeak), as shown in Figure 6. The total thickness of the newly formed weld seams is 22.31 μm (Tpeak = 325 °C), 27.64 μm (Tpeak = 340 °C), and 67.44 μm (Tpeak = 355 °C), respectively, representing deviations of −27.69 μm (Tpeak = 325 °C), −22.36 μm (Tpeak = 340 °C), and + 17.44 μm (Tpeak = 355 °C) relative to the initial solder thickness. Within the 325–355 °C range, the thickness fluctuation remains stable within −55.3% to + 34.8% of the initial thickness. The slight oscillation arises from the combined effects of non-uniform melt spreading induced by the peak temperature, the escape of adsorbed gases on the coating, and the clamping force of the fixture.

Under the conditions of Tpeak = 325 °C and Tpeak = 340 °C, the thickness results of multiple samples tested repeatedly exhibit minor differences, indicating that the weld seam thickness is relatively stable. These minor fluctuations stem from variations among different shell, cover plate, and solder ring samples; the inevitable presence of microvoids in the weld seam; slight differences in welding pressure and application position during the eutectic process; and the non-uniformity of the molten state of the solder.

When Tpeak = 355 °C, the repeated test results for the weld thickness of multiple groups of samples were 67.44 μm, 61.81 μm, 55.28 μm, 84.93 μm, 76.77 μm, 52.17 μm, 53.49 μm, 52.61 μm, 83.98 μm, and 81.73 μm, with an average of 67.02 μm and a range R of 32.76. The data span is extremely large (covering ±49% of the mean) and exhibits a bimodal distribution. The variance σ^2^ is 169.02, the standard deviation σ is 13.00, and the coefficient of variation CV is 19.40%, indicating high data volatility and a degree of dispersion far exceeding the engineering acceptable range (CV < 10%). This suggests that Tpeak = 355 °C provides excessive welding energy input, rendering the welding state uncontrollable. Analysis reveals that a large amount of gas adhering to the surface of the package and cover, as well as gas hidden in coating cracks and defects, escapes from the substrate under higher temperatures, generating numerous bubbles. Simultaneously, the high temperature induces more vigorous movement of the molten solder, drawing in substantial amounts of external gas into the molten solder. Moreover, this vigorous movement counteracts the limited clamping force provided by the sealing fixture, disrupting the balance of bubble expulsion in the weld seam by the welding pressure, making it difficult for newly generated bubbles to escape from the weld seam. These bubbles disperse in the molten solder and form weld seam voids upon solidification, not only manifesting as the weld seam thickness exceeding the initial solder thickness but also having a fatal impact on welding reliability. When the welding peak temperature exceeds the 355 °C threshold, bubble growth kinetics exhibit an accelerated evolution trend, further inducing non-steady-state evolution of the welding interface structure and a substantial increase in process uncertainty.

In conclusion, selecting the process temperature that forms a narrower weld seam (Tpeak = 325 °C or Tpeak = 340 °C) is of positive significance for reducing the void rate and improving reliability.

### 4.3. Correlation Mechanism Between Peak Temperature and IMC Reliability

**Figure 7:**
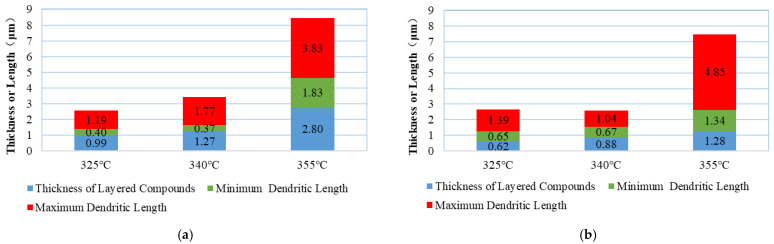
Thickness of layered compounds and length of dendrites. (**a**) Upper end of weld seam and (**b**) lower end of weld seam.

Experimental results demonstrate that the morphological evolution of IMCs at the interface between the Ni coating and Au-Sn solder is highly sensitive to the peak temperature. The thermodynamic–dynamic coupling mechanism is illustrated in Figure 7.

(1) Morphological characteristics of IMCs at the upper end of the weld seam

At Tpeak = 325 °C, the thickness of layered IMCs is 0.99 μm, which is relatively low (as shown in Figure 7a). At Tpeak = 340 °C, the thickness increases to 1.27 μm, representing a 28.3% increase compared to Tpeak = 325 °C. When Tpeak rises to 355 °C, the thickness significantly increases to 2.80 μm, nearly three times the value at Tpeak = 325 °C. These data indicate that at Tpeak = 325 °C and Tpeak = 340 °C, the thickness of layered IMCs remains within an appropriate range, adhering to the Ni coating surface and inhibiting Ni diffusion into the solder region. Generally, IMCs with thicknesses in the range of 0.5–1.5 μm achieve the optimal balance between strength and reliability. However, at Tpeak = 355 °C, excessive growth of interfacial IMCs leads to increased brittleness, compromising their reliability.

The minimum dendrite length varies with temperature as follows: 0.4 μm (Tpeak = 325 °C), 0.37 μm (Tpeak = 340 °C), and 1.83 μm (Tpeak = 355 °C), increasing more than threefold. The maximum dendrite length varies with temperature as follows: 1.19 μm (Tpeak = 325 °C), 1.77 μm (Tpeak = 340 °C), and 3.83 μm (Tpeak = 355 °C), increasing by 2–2.5 times. These data suggest that at appropriate temperatures, dendrites reach a certain length, and their three-dimensional structure penetrates into the weld seam from the interface, forming an “anchoring effect” and achieving mechanical interlocking with the weld seam area. This enhances interfacial shear strength compared to layered compounds. Additionally, these dendritic structures absorb thermal cycling and thermal shock stresses during product service, delaying thermal mismatch and delamination effects at the interface. However, excessively long dendrites are prone to stress concentration and fracture. IPC-7095D recommends controlling the aspect ratio of dendrites within the range of 3–5. At Tpeak = 355 °C, needle-like dendrites with an aspect ratio of 15 appear in large quantities. Moreover, the growth angle of dendrites becomes less predictable, with significant increases in dendrites growing at angles of 45–60° and perpendicular to the Ni coating compared to the original 75–90° orientation. Under high-temperature conditions, the changes in aspect ratio and growth orientation compromise the strength of the welding interface.

(2) Morphological Characteristics of IMCs at the Lower End of the Weld Seam

At Tpeak = 325 °C and Tpeak = 340 °C, the thickness of layered IMCs is 0.62 μm and 0.88 μm, respectively, both within an appropriate range (as shown in Figure 7b). At Tpeak = 350 °C, the thickness increases to 1.28 μm, doubling and approaching the maximum thickness for reliable interfacial compounds.

The minimum dendrite length varies with temperature as follows: 0.65 μm (Tpeak = 325 °C), 0.67 μm (Tpeak = 340 °C), and 1.34 μm (Tpeak = 355 °C), increasing nearly twofold. The maximum dendrite length varies with temperature as follows: 1.39 μm (Tpeak = 325 °C), 1.04 μm (Tpeak = 340 °C), and 4.85 μm (Tpeak = 355 °C), increasing more than threefold.

Based on the above analysis, it is concluded that at Tpeak = 325 °C and Tpeak = 340 °C, interfacial metal compounds exhibit higher reliability.

### 4.4. Regulation Mechanism of Peak Temperature on Ni Element Diffusion Behavior

**Figure 8:**
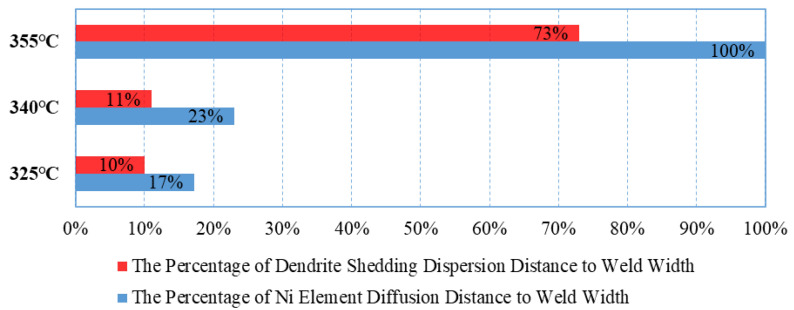
Diffusion distance of the Ni element into the weld seam.

The diffusion of Ni into Au-Sn solder is a critical step in the formation of ternary compounds in the Au-Sn/Ni system. Ni and AuSn transition layers form a superlattice ordered solid solution [15]. The diffusion behavior of Ni in this system exhibits significant temperature dependence, involving both thermal diffusion and dendrite peeling diffusion mechanisms (as shown in Figure 8).

At Tpeak = 325 °C and Tpeak = 340 °C, thermally driven diffusion dominates, causing Ni atoms to accumulate at the interface, where dendrites grow along the interface, occupying 10–11% of the weld seam width. Ni is not easily detectable macroscopically in the central region of the weld seam. At Tpeak = 355 °C, the diffusion mechanism shifts, with dendrite peeling diffusion becoming dominant. Micron-sized particles (1–5 μm) generated by the fracture of (Ni, Au)_3_Sn_2_ dendrites diffuse into the weld seam in a tide-like manner. This large-scale diffusion reaches up to 52% of the weld seam width, with the farthest distance reaching 73%, forming localized Ni-rich zones. The molar quantity of Ni in these enrichment zones is similar to that in (Ni, Au)_3_Sn_2_ at the interface. However, this diffusion exhibits significant directionality, being observed only at the upper end of the weld seam, with no large-scale shedding or diffusion from the bottom to the center of the weld seam. Currently, there is no direct experimental evidence or prior literature addressing this observation. Our analysis suggests that this may result from the combined effect of solder melt dynamics and welding pressure within the sealed region. Specifically, the micro-stress at the lower end of the weld consistently exceeds that at the upper end. These variations in micro-stress distribution are likely responsible for the observed differences in weld morphology.

Au-Sn can form a good eutectic interface, but uncertainties increase when additional reactants are involved. Therefore, conditions of Tpeak = 325 °C and Tpeak = 340 °C should be preferred to avoid excessive Ni diffusion.

### 4.5. Investigation into the Correlation Mechanism Between Aging Conditions and Intermetallic Compound (IMC) Growth

**Figure 9:**
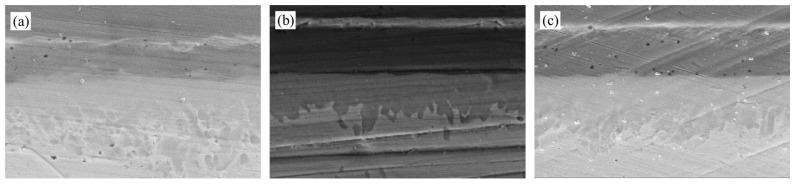
State of IMCs after aging in air: (**a**) 125 °C; (**b**) 150 °C; (**c**) 175 °C.

**Figure 10:**
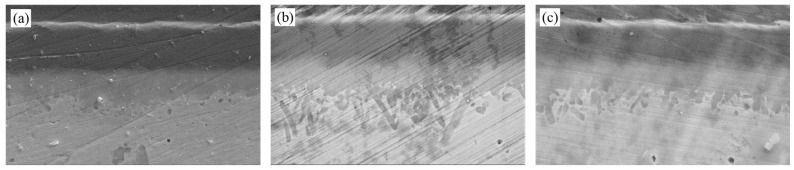
State of IMCs after aging in an argon atmosphere: (**a**) 125 °C; (**b**) 150 °C; (**c**) 175 °C.

Based on the reliability requirements for IMC thickness specified in Chinese Military Standard (GJB) [16], a gradient aging experiment (125 °C/150 °C/175 °C × oxygen/argon dual atmosphere × 600 h) was conducted to systematically investigate the cooperative effects of environmental temperature and atmosphere on IMC growth (as shown in Table 2). Figure 9 and Figure 10 illustrate the IMC structures of samples aged in air and argon.

In an oxygen environment, the thickness of interfacial IMCs after aging approaches 4.5 μm, while in an argon environment, it approaches 3.3 μm. The growth rate of IMCs in an oxygen environment is significantly higher than in an argon environment due to the promoting effect of oxidation on diffusion. Oxidation accelerates IMC growth by increasing defect density and diffusion coefficients, whereas the argon protective atmosphere effectively inhibits excessive IMC growth, resulting in final IMC thicknesses that are 26.7–37.5% lower under the same temperature conditions.

### 4.6. Airtightness Testing

According to Chinese Military Standard (GJB) [16], the sealing performance of the DPC damper substrate was tested. The applied pressure PE was 517 ± 15 kPa, the minimum pressurization time t1 was 5 h, and the leakage criterion limit value R1 was 3 × 10^−3^ (Pa·cm^3^)/s. Fifty samples were prepared for the sealing performance test. The results showed that all devices passed the sealing test, indicating that the gold-tin sealed three-dimensional DPC substrate provides excellent air-tightness.

## 5. Conclusions

Through morphological analysis of the weld seam, we have confirmed that the high-metal-content DPC three-dimensional substrate exhibits excellent Au-Sn sealing capabilities. The dominant reaction pathway involves Ni interacting with Au-Sn to form (Ni, Au)_3_Sn_2_ and Au_5_Sn intermetallic compounds. However, upon completion of the sealing process, the reaction does not proceed to full completion; instead, a coexistence of (Ni, Au)_3_Sn_2_, Au_5_Sn, and residual AuSn phases is observed. Reliability assessments confirm that this partial reaction is optimal for maintaining structural integrity. Ni effectively functions as a diffusion barrier layer. Nevertheless, Ni can still migrate into the weld center via dendritic fragmentation and locally form secondary δ(Ni) and ζ’(Ni) phases. This indicates that the weld interface constitutes a complex, non-uniform, and asymmetric microstructure comprising multiple coexisting intermetallic compounds. A peak welding temperature range of Tpeak = 325 °C to 340 °C is identified as optimal, under which the weld morphology, weld width, and Ni diffusion level reach ideal states, ensuring satisfactory hermeticity of the device. Exceeding this temperature range leads to excessive thermal input, resulting in process instability and progressive deviation of the weld interface from the desired configuration. The root cause lies in the accelerated kinetics of bubble growth under elevated temperatures, where entrapped gas solidifies within the molten solder, forming voids. This phenomenon not only increases the weld thickness beyond the initial solder layer but also significantly compromises welding reliability. Interfacial intermetallic compounds tend to thicken with increasing aging temperature. However, long-term aging tests demonstrate that IMC growth remains within a controllable range. Comparative analysis further reveals that an argon atmosphere yields a more stable and favorable interface.

## Figures and Tables

**Table 1:** Molar quantity ratio of typical compound exploration points.

Component	Ni	Au	Sn
(Ni, Au)_3_Sn_2_	34.52%	27.38%	38.10%
	20.86%	36.50%	42.64%
AuSn	-	50.72%	49.28%
		50.39%	49.61%
Au_5_Sn	-	85.19%	14.81%
		79.87%	20.13%

**Table 2:** Aging test conditions and thickness growth of IMCs.

No.	Temperature/°C	Atmosphere	Time/h	Initial IMC Thickness/μm	IMC Thickness After Aging/μm	Thickness Growth Rate/%
1#	125	air	600	3	4	33.3
2#	150			2.45	4	63.3
3#	175			3	4.5	50.0
4#	125	Ar		2	2.5	25
5#	150			2.45	3.3	34.7
6#	175			2.9	3.3	13.8

## Data Availability

The original contributions presented in this study are included in the article. Further inquiries can be directed to the corresponding authors.

## References

[1] Lu Q., Liu K., Qiao Z., Liu L., Gao L. (2021). Research Status and New Progress of Ceramic Substrate. Semicond. Technol..

[2] Cheng H., Chen M., Luo X., Peng Y., Liu S. (2019). Ceramic Substrate for Electronic Packaging. Adv. Ceram..

[3] Yang Y., Li Y., Zheng W., Yu F., Yang H., Yi X., Wang J., Li J. (2020). High-reflection Al-plated DPC ceramic substrate for AlGaN-based DUV LED packaging. Chin. J. Liq. Cryst. Disp..

[4] Wang K., Jiang B., Gan D. (2015). Orientation relationships of Au_5_Sn/Au interfaces and formation sequence of Au_5_Sn and AuSn at the Au/Sn interface. Thin Solid Film..

[5] Wei X., Zhu X., Wang R. (2017). Growth behavior and microstructure of intermetallics at interface of AuSn20 solder and metalized-Ni layer. Trans. Nonferrous Met. Soc. China.

[6] Yoon J., Jung S. (2007). Investigation of interfacial reaction between Au-Sn solder and Kovar for hermetic sealing application. Microelectron. Eng..

[7] Alam M., Chan Y., Tu K. (2004). Elimination of Au-embrittlement in solder joints on Au/Ni metallization. J. Mater. Res..

[8] Yoon J., Chun H., Koo J. Au-Sn flip-chip solder bump for microelectronic and optoelectronic applications. Proceedings of the Symposium on Design, Test, Integration and Packaging of MEMS/MOEMS.

[9] Wang M., Zhu Z., Wei X., Feng Y. (2014). Interfacial reaction and shear strength of AuSn/(Ni/AlSi) joint. J. Cent. South Univ. (Sci. Technol.).

[10] Dong H.Q., Vuorinen V., Liu X. (2016). Microstructural Evolution and Mechanical Properties of Au-20wt.%Sn|Ni Interconnection. J. Electron. Mater..

[11] Peng J., Wang R., Wang M. (2017). Interfacial Microstructure Evolution and Shear Behavior of Au-Sn/NixCu Joints at 350A°C. J. Electron. Mater..

[12] Zhu Z., Li C., Liao L. (2016). Au-Sn bonding material for the assembly of power integrated circuit module. J. Alloys Compd..

[13] Zhu Z., Rengganathan V., Kao C. (2021). Grain Boundary Diffusion of Ni through Au-Doped Ni_3_Sn_2_ Intermetallic Compound for Technological Applications. J. Electron. Mater..

[14] Liu X., Kinaka M., Takaku Y. (2005). Experimental investigation and thermodynamic calculation of phase equilibria in the Sn-Au-Ni system. J. Electron. Mater..

[15] Ding F., Wang Q., Liang C., Zhang Y. (2025). Diffusion behavior and microstructural evolution of bonding interface between AuSn20 and tungsten-copper alloy. Solder. Surf. Mt. Technol..

[16] (2021). Standard for Test Methods for Mechanical Properties on Ordinary Concrete.

